# Sinus Venosus Atrial Septal Defect Complicated by Eisenmenger Syndrome and the Role of Vasodilator Therapy

**DOI:** 10.1155/2016/8164923

**Published:** 2016-11-14

**Authors:** Amornpol Anuwatworn, Maheedhar Gedela, Edgard Bendaly, Julia A. Prescott-Focht, Jimmy Yee, Richard Clark, Orvar Jonsson

**Affiliations:** ^1^Sanford Cardiovascular Institute, University of South Dakota Sanford School of Medicine, Sioux Falls, SD, USA; ^2^Department of Internal Medicine, University of South Dakota Sanford School of Medicine, Sioux Falls, SD, USA; ^3^Department of Pediatrics, University of South Dakota Sanford School of Medicine, Sioux Falls, SD, USA; ^4^Department of Radiology, University of South Dakota Sanford School of Medicine, Sioux Falls, SD, USA

## Abstract

Sinus venosus atrial septal defect is a rare congenital, interatrial communication defect at the junction of the right atrium and the vena cava. It accounts for 5–10% of cases of all atrial septal defects. Due to the rare prevalence and anatomical complexity, diagnosing sinus venous atrial septal defects poses clinical challenges which may delay diagnosis and treatment. Advanced cardiac imaging studies are useful tools to diagnose this clinical entity and to delineate the anatomy and any associated communications. Surgical correction of the anomaly is the primary treatment. We discuss a 43-year-old Hispanic female patient who presented with dyspnea and hypoxia following a laparoscopic myomectomy. She had been diagnosed with peripartum cardiomyopathy nine years ago at another hospital. Transesophageal echocardiography and computed tomographic angiography of the chest confirmed a diagnosis of sinus venosus atrial septal defect. She was also found to have pulmonary arterial hypertension and Eisenmenger syndrome. During a hemodynamic study, she responded to vasodilator and she was treated with Ambrisentan and Tadalafil. After six months, her symptoms improved and her pulmonary arterial hypertension decreased. We also observed progressive reversal of the right-to-left shunt. This case illustrates the potential benefit of vasodilator therapy in reversing Eisenmenger physiology, which may lead to surgical repair of the atrial septal defect as the primary treatment.

## 1. Introduction

Sinus venosus atrial septal defect (SVASD) is one of the major categories of atrial septal defect (ASD). SVASD was first reported in 1858 and accounts for 5–10% of cases of ASD [1, 2]. It usually coexists with partial anomalous pulmonary venous return (PAPVR), which leads to additional left-to-right shunting. SVASD is generally unrecognized until the fifth decade of life when it presents as right heart failure and/or pulmonary hypertension [3]. SVASDs are usually located near the superior or inferior vena cava entry into the right atrium. This interatrial communication leads to volume overload, right-sided chamber dilation, and ultimately pulmonary hypertension [1, 3]. In this paper, we present a case of pulmonary artery hypertension and Eisenmenger syndrome due to a superior form of SVASD. We will also discuss the beneficial role of vasodilator therapy in reversing the right-to-left shunt, which may stabilize a patient for future surgery.

## 2. Case Report

A 43-year-old Hispanic woman was transferred to our facility with hypoxia following laparoscopic hysteroscopy and myomectomy. She reported experiencing exertional dyspnea for four years. She also disclosed a history of cardiomegaly that was discovered on both a chest X-ray and through transthoracic echocardiography (TTE) nine years ago at another hospital, and she had been treated for postpartum cardiomyopathy. Physical examination revealed the following: heart rate, 90 beats/minute; blood pressure, 126/85 mmHg; respiratory rate, 24 breaths/minute; and oxygen saturation, 88% on 15 liters of oxygen by nasal cannula. The patient had digital clubbing, right ventricular lift, and a grade 2/6 systolic ejection murmur at the second left intercostal space.

**Figure 1:**
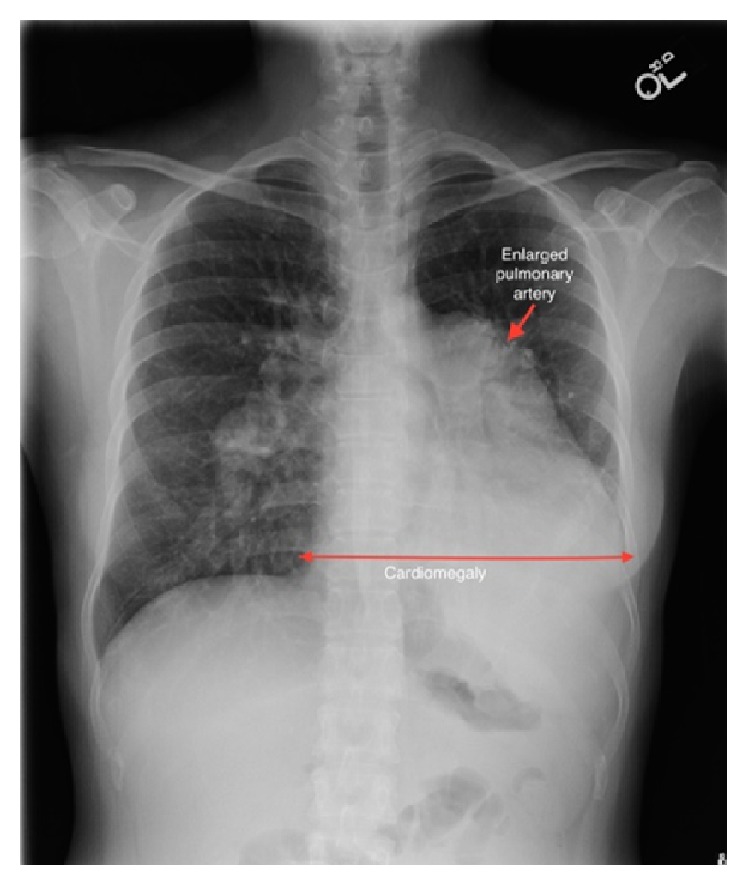
A chest radiograph shows a markedly enlarged pulmonary trunk (red arrow), enlarged central pulmonary arteries, and enlargement of the cardiac silhouette.

**Figure 2:**
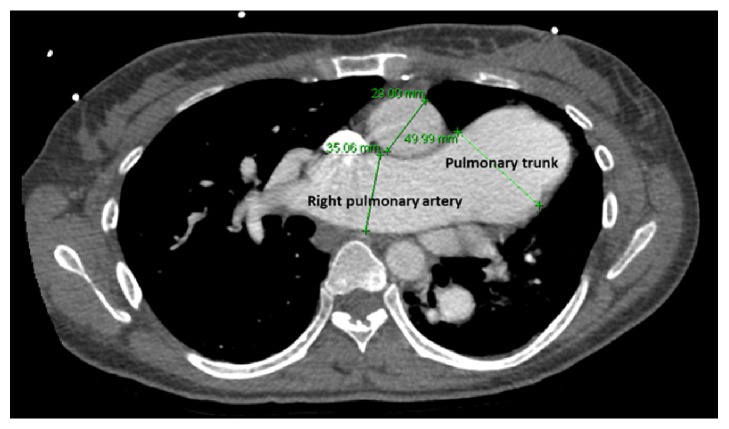
Computed tomographic angiography of the chest shows a markedly dilated pulmonary trunk, at 49.99 mm (normal < 30 mm); a pulmonary trunk-to-aorta ratio of 1.7 (normal < 1.0); and an enlarged right pulmonary artery, at 35.06 mm (normal < 20 mm).

**Figure 3:**
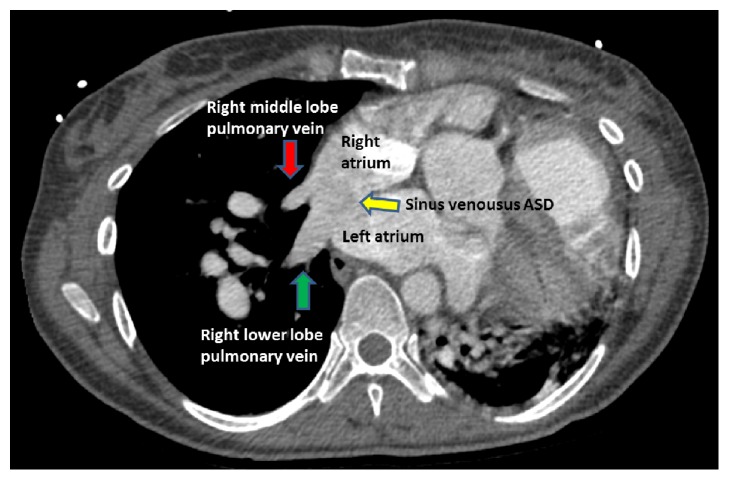
Computed tomographic angiography of the chest shows the right middle lobe vein (red arrow) draining into the right atrium (partial anomalous pulmonary venous return) and the right lower lobe vein (green arrow) entering the heart at the junction of the right and the left atria at the level of a large atrial septal defect (yellow arrow).

**Figure 4:**
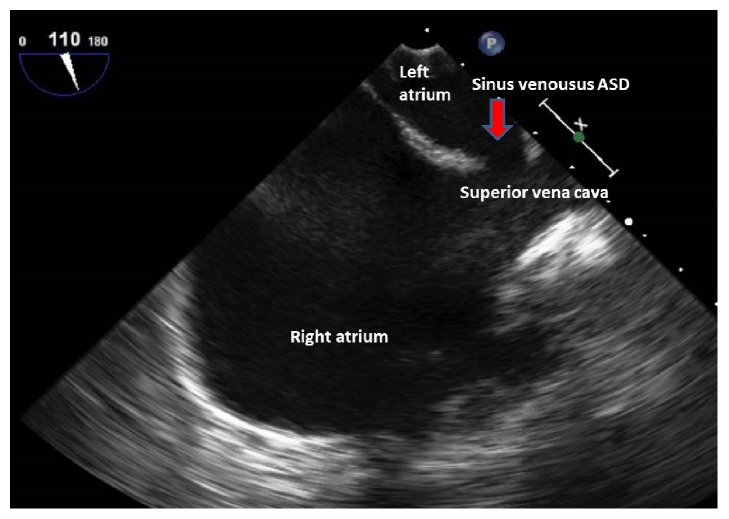
A bicaval view via a transesophageal echocardiogram shows communication (red arrow) between the left atrium and the superior vena cava entry.

An electrocardiogram (EKG) revealed right axis deviation, a right ventricular hypertrophy, and a left atrial enlargement. A chest X-ray showed an enlarged cardiac silhouette and significantly enlarged pulmonary trunk and central pulmonary arteries (Figure 1). Computed tomographic angiography (CTA) of the chest was performed to rule out a pulmonary embolism in the emergency room. This study showed markedly enlarged central pulmonary arteries suggestive of pulmonary arterial hypertension (PAH) (Figure 2). In addition, the CTA showed cardiomegaly with marked right heart chamber enlargement, right ventricular wall hypertrophy, a large atrial septal defect (ASD), and right upper and middle lobe PAPVR (Figure 3). TTE displayed a normal left ventricular ejection fraction, a severely dilated right atrium and ventricle, and moderate pulmonic and tricuspid regurgitation. The right ventricular systolic pressure was 67 mmHg. Notably, there was no interatrial shunt revealed by color Doppler. A transesophageal echocardiogram (TEE) revealed a moderately sized SVASD (Figure 4) and a bidirectional atrial shunt with saline contrast.

In order to assess hemodynamics, a right heart catheterization was performed, which showed the mean pulmonary artery pressure to be 46 mmHg (normal value 14 ± 3.3 mmHg). Pulmonary vascular resistance (PVR) was calculated at 11.1 Wood units·m^2^ which is consistent with PAH (defined as a PVR > 3 Wood units·m^2^). The ratio of the PVR to the systemic vascular resistance (SVR) was elevated, at 0.6 : 1. The intracardiac shunting *Q*_*P*_/*Q*_*S*_ was 1.1 : 1, which indicates left-to-right shunting. This ratio takes into consideration the left-to-right shunting from the anomalous pulmonary veins draining into the right atrium. This is demonstrated by the saturation step up between the high superior vena cava (SVC) (65%) and the right atrium (71%). However, shunting was right-to-left across the ASD, as demonstrated by a systemic oxygen saturation of 83%. These findings were consistent with Eisenmenger physiology. After administration of inhaled nitric oxide at 40 parts per million (40 ppm) and 100% FiO_2_, the patient's pulmonary vascular bed was slightly responsive to vasodilation. The PVR decreased to 9 Wood units·m^2^, and the ratio of PVR to SVR was 0.37 : 1. Although there was a slight improvement in the *Q*_*P*_/*Q*_*S*_ to 1.44 : 1, right-to-left shunting across the ASD persisted. This was demonstrated by a low, systemic arterial oxygen partial pressure (paO_2_) of 165 mmHg, compared to a pulmonary venous pAO_2_ of 353 mmHg. In this case, CTA was initially performed to exclude pulmonary embolism in the ER. Cardiac magnetic resonance imaging (CMR) was not performed since we obtained enough anatomical and hemodynamic information from CTA, TEE, and right heart catheterization. If CTA had not been done in the ER, then we would have done CMR because it is a better imaging modality to evaluate hemodynamics of the ASD.

Based on the patient's history, physical examination, EKG, and imaging findings, she was diagnosed with PAH and Eisenmenger syndrome secondary to a superior form of SVASD. Given her hemodynamic response to vasodilation therapy, she was treated with Ambrisentan and Tadalafil to reduce pulmonary vascular resistance and right ventricular volume overload and to reverse shunting. After treatment was initiated, the patient's clinical symptoms improved. She was able to walk greater distances and climb stairs. Her SaO_2_ increased from 87% on room air to 92%. After one year of therapy, she was taken to the cardiac catheterization lab for a hemodynamic assessment. At baseline, her right heart catheterization revealed a PVR of 6.6 Wood units·m^2^, with a pulmonary/systemic vascular resistance (*R*_*p*_/*R*_*s*_) ratio of 0.61 and an overall *Q*_*P*_/*Q*_*S*_ of 0.93 : 1. After administration of inhaled nitric oxide at 40 ppm and 100% FiO_2_, the PVR was 5 Wood units·m^2^ with an *R*_*p*_/*R*_*s*_ ratio of 0.37 : 1, and the *Q*_*P*_/*Q*_*S*_ was 2.84 : 1. Overall, the PVR improved, and the *Q*_*P*_/*Q*_*S*_ with vasodilation therapy favored a larger left-to-right shunt.

## 3. Discussion

SVASD is a rare congenital, interatrial communication at the junction of the right atrium and the superior vena cava entry (superior form) or inferior vena cava entry (inferior form). The superior SVASD comprises 5–10% of cases of ASD [1, 2]. The interatrial left-to-right shunting leads to right ventricular volume overload and increases in pulmonary blood flow and pulmonary vascular resistance [1, 3]. The left-to-right shunting is significant when the ratio of *Q*_*P*_/*Q*_*S*_ is greater than 1.5 : 1, or when a patient develops dilation of the right heart chambers [2]. The right ventricular volume overload results in right ventricular dilation, pulmonary vascular modeling, pulmonary hypertension, and ultimately Eisenmenger physiology, which is an irreversible right-to-left shunt [3] as illustrated in this case.

Due to its rarity, SVASD may go unnoticed and the diagnosis may be delayed. Up to 75% of patients with an uncorrected ASD present with dyspnea by the fifth decade of life [3]. In addition, atrial fibrillation or flutter, decompensated right heart failure, paradoxical embolus, and/or transient ischemic attack are associated with ASDs [2]. Unexplained dilation of the right atrium or right ventricle should prompt clinicians to do further evaluations for intracardiac shunts [1–3]. TTE is usually the first step in the workup for SVASD; however, only 12% of superior SVASDs can be visualized [3]. The diagnosis of SVASD is more challenging than other forms of ASDs due to its complex anatomy and may require advanced imaging such as TEE, magnetic resonance imaging or CTA [4]. TEE is more sensitive than TTE in visualizing SVASDs. TEE is also helpful in identifying PAPVR and assessing the size and position of an ASD [5]. Furthermore, the superior form of SVASD usually coexists with PAPVR and leads to additional left-to-right shunting [2]. SVASD is accompanied by PAPVR in 95–97% of patients [3]. The sensitivity of CTA of the chest in detecting PAPVR among patients with suspected SVASD is 100%, and specificity is also 100% [4].

Surgical repair is only the treatment of choice for SVASD to prevent progressive right heart dysfunction [5]. Percutaneous closure is not feasible due to the inadequate tissue margins required to securely join the closure device in addition to the risk for systemic and pulmonary venous inflow obstruction [2, 3, 5]. According to the 2008 American College of Cardiology/American Heart Association guidelines, ASD closure is not recommended in patients with severe, irreversible PAH because of the risk of acute right heart failure after the closure [1, 3]. However, there has been a case reported of the successful closure of an ASD with irreversible PAH after a hemodynamic intracardiac shunt was reversed by vasodilator therapy [6]. Schwerzmann et al. reported the benefit of using intravenous prostacyclin to reduce PAH in a patient with a secundum ASD complicated by PAH and Eisenmenger physiology. After one year of intravenous prostacyclin treatment, pulmonary arterial pressure and PVR decreased and the patient underwent a successful percutaneous closure of the ASD. The patient was eventually weaned off of intravenous prostacyclin and continued to take oral Bosentan [6].

In this case, Eisenmenger physiology resulted from untreated SVASD, not secundum ASD like in Schwerzmann et al.'s report. It is of paramount importance that clinicians consider ASD in the differential diagnoses of patients with chronic dyspnea on exertion, hypoxemia, enlarged right atrium, and enlarged right ventricles so the diagnosis can be made early and late complications can be prevented, such as Eisenmenger's physiology in this case. In addition, our case demonstrates the favorable effect of vasodilator therapy in a SVASD patient with Eisenmenger syndrome. The patient was evaluated for surgery to make the SVASD smaller and to correct PAPVR. The intent was to decrease right ventricular volume overload but without complete closure so as to allow a pop-off valve in the event of right-to-left shunting. If the patient's pulmonary arterial hypertension improves after the surgery, complete percutaneous closure of the defect will be a consideration.
